# Extending coverage to informal sector populations in Kenya: design preferences and implications for financing policy

**DOI:** 10.1186/s12913-017-2805-z

**Published:** 2018-01-09

**Authors:** Vincent Okungu, Jane Chuma, Stephen Mulupi, Diane McIntyre

**Affiliations:** 1 Institute of Healthcare Management, Strathmore University, Nairobi, Kenya; 2 Pharmaccess Foundation, Nairobi, Kenya; 3 KEMRI-Wellcome Trust Research Programme, Nairobi, Kenya; 4 African Population and Health Research Centre, Nairobi, Kenya; 5 Health Economics Unit, University of Cape Town, Cape Town, South Africa

**Keywords:** Universal health coverage, Informal sector, Prepayment designs

## Abstract

**Background:**

Universal health coverage (UHC) is important in terms of improving access to quality health care while protecting households from the risk of catastrophic health spending and impoverishment. However, progress to UHC has been hampered by the measures to increase mandatory prepaid funds especially in low- and middle-income countries where there are large populations in the informal sector. Important considerations in expanding coverage to the informal sector should include an exploration of the type of prepayment system that is acceptable to the informal sector and the features of such a design that would encourage prepayment for health care among this population group. The objective of the study was to document the views of informal sector workers regarding different prepayment mechanisms, and critically analyze key design features of a future health system and the policy implications of financing UHC in Kenya.

**Methods:**

This was part of larger study which involved a mixed-methods approach. The following tools were used to collect data from informal sector workers: focus group discussions [*N* = 16 (rural = 7; urban = 9)], individual in-depth interviews [*N* = 26 (rural = 14; urban = 12)] and a questionnaire survey [*N* = 455(rural = 129; urban = 326)]. Thematic approach was used to analyze qualitative data while Stata v.11 involving mainly descriptive analysis was used in quantitative data. The tools mentioned were used to collect data to meet various objectives of a larger study and what is presented here constitutes a small section of the data generated by these tools.

**Results:**

The findings show that informal sector workers in rural and urban areas prefer different prepayment systems for financing UHC. Preference for a non-contributory system of financing UHC was particularly strong in the urban study site (58%). Over 70% in the rural area preferred a contributory mechanism in financing UHC. The main concern for informal sector workers regardless of the overall design of the financing approach to UHC included a poor governance culture especially one that does not punish corruption. Other reasons especially with regard to the contributory financing approach included high premium costs and inability to enforce contributions from informal sector.

**Conclusion:**

On average 47% of all study participants, the largest single majority, are in favor of a non-contributory financing mechanism. Strong evidence from existing literature indicates difficulties in implementing social contributions as the primary financing mechanism for UHC in contexts with large informal sector populations. Non-contributory financing should be strongly recommended to policymakers to be the primary financing mechanism and supplemented by social contributions.

**Electronic supplementary material:**

The online version of this article (10.1186/s12913-017-2805-z) contains supplementary material, which is available to authorized users.

## Background

Since the 58^th^ World Health Assembly, many low- and-middle-income countries (LMICs) are reforming their health systems for universal health coverage (UHC). UHC has two key goals: financial risk protection and access to needed care. Implicit are objectives related to equity, quality of services and broader social protection [1]. The World Health Organization (WHO) recommends greater use of mandatory prepayment mechanisms including general government revenue (tax funding) and social health insurance (SHI). Both tax funding and SHI can mobilise substantial resources, create income and risk cross-subsidies and benefit from economies of scale. Large risk pools are more financially secure and promote equity in financing and access to health care across socio-economic groups [2, 3].

The need for UHC is well documented. However, the measures to increase mandatory prepaid funds to progress toward UHC are faced with a number of challenges including inefficiencies in revenue collection and use of health resources, inequitable access and utilisation of health services and large populations working in the informal sector [4–6].

The ILO & WTO [7] indicates that up to 65% of the total population in low- and middle-income countries (LMICs) work in the informal sector. In Kenya, informal sector workers constitute about 80% of the total workforce [8, 9]. The sector is characterized by low and irregular incomes which make prepaying for health care difficult. As a result, existing prepayment systems including funding from government revenue and premium contributions in Kenya and many LMIC tend to exclude informal sector workers because either the funds are inadequate (in the case of funding from general government revenue) or they are too poor to pay for insurance premiums [3]. Moreover SHI schemes historically have focused on the formal sector workers because it is relatively easy by law to enforce mandatory contributions through salary deductions [3, 5].

A number of developing countries are reforming their health systems for UHC. In Kenya, financing reforms for UHC are underway and the process of finalizing a health financing strategy (HFS) has been going on for more than two years. The draft HFS proposes a contributory health insurance model as the main health financing strategy for Kenya where both formal and informal sector workers contribute premiums to a scheme, and the government subsidizes premiums for the poor and other vulnerable groups.

The contributory policy approach to financing UHC in Kenya is a technocrat-led top-down strategy with limited public participation and partly informed by the assumption that there are sufficient financial resources in the informal sector to support the UHC agenda. There is limited evidence to suggest that the views of informal sector workers regarding their preferred prepayment design were put into consideration. Thornton et al. [10] reiterate the scarcity of evidence on the best possible design of a prepayment system that targets the informal sector in terms of ability to achieve high coverage or to increase utilisation of quality health services across different contexts. Extending coverage to the informal sector through an inclusive process increases their participation in decision-making processes and break away from exclusion [11, 12], for example from prepayment systems. This study fills this gap in evidence by exploring informal sector workers’ preference of health financing mechanisms for UHC. It presents the views of informal sector workers on the design of future prepayment mechanisms and how such a design could drive up an all-inclusive population coverage in Kenya. The aim of this paper is to illustrate the preferences of the informal sector in designing a prepayment mechanism with the aim of expanding population and service coverage to achieve UHC.

## Methods

Data presented in this paper were part of a larger study whose aim was to contribute to national and international policy debates on UHC in contexts with large informal sector populations. The study was conducted in two counties: Nyeri, a rural county that relies predominantly on agriculture; and Mombasa, the second largest city in Kenya. The combination of an urban and a rural county provided an opportunity to compare and contrast the views of agricultural and non-agricultural informal workers. In addition, existing studies on the informal sector in Kenya including [13–15], have all been conducted in Nairobi, the largest urban setting in Kenya. Nyeri County was purposively selected based on the fact that it has a long history of community based health insurance (CBHI) schemes. There was clear indication that the population was potentially aware of health insurance issues and hence their insights and experiences were important for this study, particularly in relation to exploring alternative prepayment mechanisms.

**Fig. 1:**
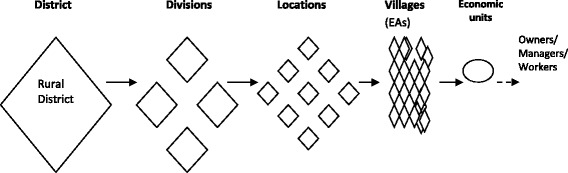
Five-stage sampling in the rural study site

**Fig. 2:**
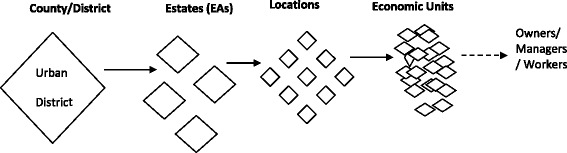
Four-stage sampling in the urban study site

A stratified five-stage and four-stage sample designs were applied in the rural and urban sites respectively, to arrive at primary sampling units (PSU) (Figs. 1 & 2). Expert opinion was used to select areas where informal sector entities were most active.

The rural site was stratified along existing administrative zones comprising of divisions, locations and villages. The village, as the smallest sampling unit, was selected as the Enumeration Area (EA). Since the divisions were on the whole, homogenously engaged in subsistence agriculture, one division with a large market was purposively selected to obtain a mix of the agricultural and non-agricultural informal sector activities in rural areas. From this division, a list of locations and villages were selected from which economic entities were mapped and randomly selected. Households represented economic units (farms) in the agricultural informal sector. The owners or managers working within the agricultural informal sector were interviewed at home except for the few who were found at the farms and preferred to be interviewed on site.

In the urban site, a list of estates was obtained from the town authorities. The estates including the central business district (CBD) constituted four purposively selected enumeration areas (EAs) for the urban area. The four EAs were selected because they had the largest concentration of informal sector entities and so provided the best opportunity to capture a large diversity in the type and size of informal sector entities. From the four urban EAs, a list of locations was obtained from which economic entities were mapped and randomly selected.

A representative sample size for quantitative component of the study was calculated using the following formula (+30% to account for non-response):

$$ N=\frac{{\left({Z}_{crit}\right)}^2p\left( 1-p\right)}{D^2}= 499\left(\approx 500\right)\kern0.5em economic\kern0.5em entities $$

-where ***N*** represented the sample size of the study group, the ***Z***_***crit***_ value is = 1.96, *p* (=0.5) is based on previous studies [16] and represents the pre-study estimate of the proportion to be measured (that is, the proportion of informal sector entities that have financial potential to prepay for health care) and ***D*** (=0.05) is the total width of the expected confidence interval (CI). The urban sample consisted of 350 economic entities and 150 for the rural area. The allocation was based on a mapping of informal sector entities which showed that for every informal sector entity in the rural site, there were about 2.3 entities in the urban site.

For the qualitative component, a conservative estimate was given as 12 focus group discussions (FGDs) stratified by gender, 17 key informant interviews at community level and five policy level in-depth interviews. Purposive and snow-balling sampling strategies were used to collect the qualitative data from key informants. Data were collected to saturation.

Phase 1 of data collection involved mapping informal sector entities in terms of geographical location and types of entities (*N* = 2721). Phase 2 included FGDs and in-depth interviews (IDIs). A total of 16 FGDs (Rural = 7, Urban = 9) and 16 community level IDIs (Rural = 14, Urban = 12 and Policy Level = 9) were conducted. Phase 3 was an interviewer administered questionnaire survey which achieved 91% response rate [*N* = 455 out of 500; Rural = 64 females, 65 males; Urban = 92 females, 234 males)]. The variety of data collection tools mentioned above were used to collect data to meet various objectives of the larger study and what is presented in this article constitutes a small section of the data generated by these tools. So not all the data generated from the large qualitative study were relevant to this study. The broad topics explored that were relevant to this paper included design preferences of future prepayment system including choice of a prepayment system, who should prepay for health care and management of prepaid funds for health. In particular, the study participants were asked what type of prepayment system between contributory and non-contributory mechanisms, they would prefer to be the main financing approach if all Kenyans were to receive quality health care under a universal coverage system. The preference for a dominant financing mechanism was with the knowledge that both contributory and non-contributory mechanisms operate concurrently in many health systems. The study participants were also asked to discuss who should and who should not pay for health care. The design of the benefit package was also discussed; however, the description of the benefit package was broadly presented as it is one that requires more technical considerations beyond the views of informal sector workers. Lastly, the study participants were asked to discuss how the management of a future prepaid system could be made more transparent to avoid corruption. Key issues raised from FGDs including choice of a prepayment system and payment for health care, were further explored with key informants.

Quantitative data were double-entered into predesigned data entry spread-sheets in FoxPro and transferred to Stata Version 11 for analysis. They were categorised and grouped to give a summary of results using descriptive statistics.

All qualitative data were audio recorded and translated into English where necessary. Data were organised and coded using QSR NVivo 7.0. Analysis was performed by developing a matrix of emerging categories and themes. Data from each theme or category was identified and analysed using constant comparison [17]. Concepts and themes identified from various qualitative data sources were compared. The analytical categories were then explained and interpreted in line with the research objectives and the themes developed from the data [18].

## Results

To expand population coverage to its members, key design elements were preferred by informal sector workers (See Additional files 1, 2 & 3 for tools used in data collection).

### Design element 1: Choice of financing mechanism

**Fig. 3:**
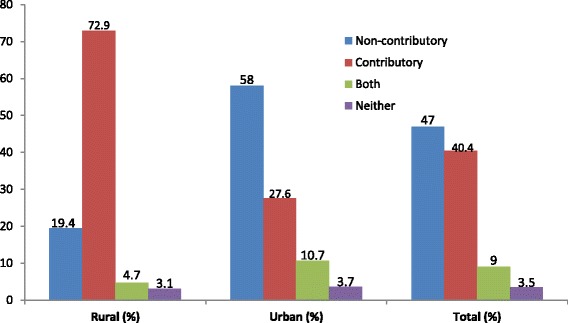
Preferred prepayment mechanisms by the informal sector

On average, 47% of the study participants, representing the largest single majority, preferred a non-contributory mechanism compared to 40% that preferred the contributory system. About 13% were indifferent (Fig. 3). There were significant differences (*P* = <0.001) between urban and rural study participants in their preferences for either a contributory or non-contributory mechanism.

An overwhelming majority (73%) of the rural area informal sector workers preferred a contributory financing approach to UHC compared to 28% in the urban area. The main reason given for their choice was familiarity with contributory schemes. The majority of study participants expressed knowledge of the NHIF and CBHI schemes. An FGD participant said: *“We are used to the schemes and they are what we would like to provide health care for all…”* (Female FGD1, Rural). Another participant added: *“We have several questions regarding a non-contributory system: how does it work? How will we know how much is collected for health care? Who will keep the money?”* (Female FGD3, Rural).

The other reason closely related to first one is that rural informal sector workers “know each other” which to them makes managing contributions transparent and regular. One FGD participant said: *“It is important that we have a local health scheme where members contribute regularly because we know each other and those in charge of managing the funds are from among ourselves….so chances of mismanaging our funds are limited”* (Male FGD5, Rural)*.*

Beyond familiarity with schemes, the theme of corruption was recurrent in all FGDs and key informant interviews in both urban and rural study sites. The general feeling among informal sector workers was that any financing mechanism that is controlled by the government is a recipe for corruption. One key informant observed: *“We do not consider ourselves as a part of this government…and so we cannot give it any more money to keep under the pretence of providing free health care because we will never get free health care or anything from the government”* (KI3, Urban). Although this information may not be applicable in a different setting, it highlights the importance of trust and the political context in advancing population and service coverage among informal sector populations.

As opposed to the rural area, more than half of the urban population (58%) preferred a non-contributory mechanism of financing health services. Study participants who favoured a non-contributory financing mechanism raised a number of reasons for their choice including the ease of implementation and revenue collection and low administrative costs. A key informant observed: *“We would like health care for all to be funded by the government because taxes are much easier to collect than insurance contributions. Only the law needs to change, which does not cost much...”* (Male KI7, Urban).

Concerns about sustainable financing also influenced preference for a non-contributory system over a contributory mechanism. This featured strongly in nine of the 12 FGDs and 14 KI interviews with most study participants stating that under a predominantly non-contributory mechanism, chances of defaulting in payment or dropping out of coverage are eliminated. A key informant had this to say: *“I would support a non-contributory system because all the taxes will be going to the government which will be expected to provide an agreed upon package of care whether we pay or not pay because it is already a deal…. But come to think of it, no one can claim to drop out of a tax-funded system because money for health care will be based on taxes on consumables, among others”* (KI3, Urban). The statement also indicates that with each citizen potentially paying directly or indirectly for health care, there would be a greater sense of entitlement to a given package of care which would make the government more accountable.

The non-contributory financing mechanism was also regarded by study participants as more politically sustainable under existing circumstances in the country. Study participants argued that with the sharp political divisions current in the country, a non-contributory mechanism would be more acceptable and sustainable financing option as it would not emphasise the low levels of national integration; i.e. there would be no information regarding which county or region has contributed how much for health care. A participant explained: *“…. There are rich counties and very poor counties and these may not like each other for purely political and ethnic reasons. If people are going to be told that their contributions could be used anywhere in the country, you will see how politicians twist everything into a mess. So to me a non-contributory approach would be the best way forward”* (Female, FGD1 Urban).

In addition, study participants reported that the non-contributory approach was relatively affordable to informal sector workers and the poor because indirect taxes are only payable upon consumption of certain goods and services. In their argument on affordability, the participants indicated a measure of equity in financing through tax-funded system. An FGD participant said: *“In one way or the other we all pay taxes directly or indirectly and the more one buys the more one pays. So I think a tax system will protect the poor who are likely to buy less of the taxable items or services”* (Male8, FGD Urban)*.* However, it is known that indirect taxes can be inequitable, particularly in high-income settings.

Informal sector workers opposed to either mechanism were mainly concerned with corruption in government and indicated that they would rather pay for health care out-of-pocket to avoid their money being embezzled. Anxiety over mismanagement of funds, distrust in the ability of the government to deliver on the agreed package of care and affordability of premiums were some of the key issues reported as potential barriers to the development of prepaid health care and UHC in Kenya.

Opinions were sought about the specific design features of the financing mechanisms including revenue collection and pooling arrangements and payment strategies. Regarding revenue collection, most study participants stated that revenue collection should involve easy means of prepayment including devolved payment units and use of wireless technology such as mobile phones and internet.

**Fig. 4:**
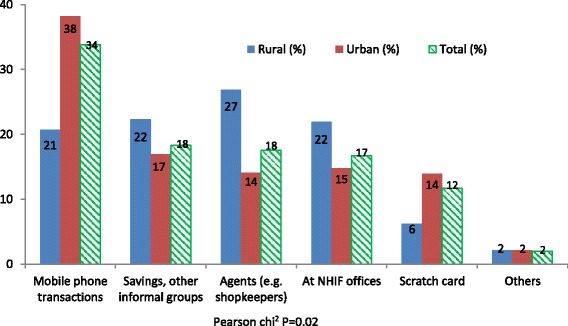
The most preferred premium payment methods (%)

Figure 4 illustrates the preferred premium payment strategies as proposed by informal sector workers. Results show significant differences in preferred payment methods between rural and urban areas (*P* = 0.02).

Rural participants preferred premium payment methods and devolved agency payment model using small scale informal businesses as agents for the insurance agency (27%) and through savings and other informal community groups (22%). In the urban area, use of mobile telephony, savings and other informal community groups were the most preferred (38% and 17%) respectively. Overall, use of mobile telephony to pay premiums was most popular (34%) followed by savings and other informal groups (18%). About 54% of informal sector workers preferred to pay their contributions on monthly basis; 19% preferred annual contributions, and 27% preferred flexible payment terms which would enable them to pay small instalments towards their monthly targets. The varying choices on the frequency of payment of premiums under a potential contributory financing mechanism, to a great extent, mirrors income inequalities in the informal sector in the sense that those who can afford to pay yearly lump-sum are those who are relatively well-off compared to those who would like to pay in small instalments spread over a month.

### Design element 2: Population and cost coverage- who gets covered, who pays?

**Fig. 5:**
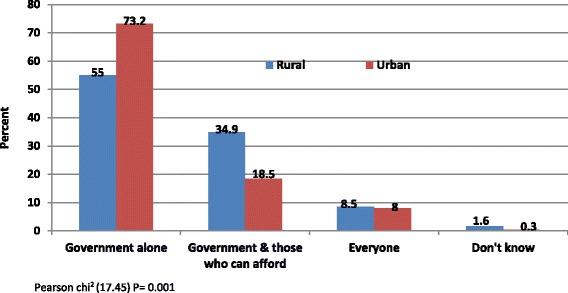
Who should pay for health care?

**Fig. 6:**
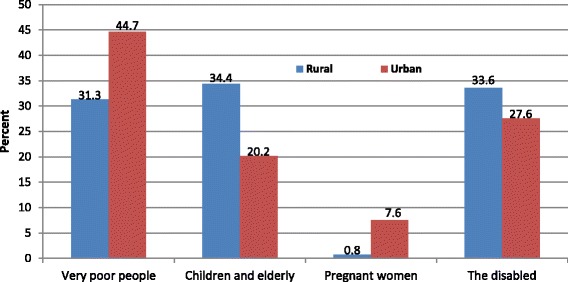
Who should not pay for health care?

Quantitative findings (Figs. 5 and 6) indicated the desire by most study participants in both sites for the government to pay for health care for everyone as a matter of priority. In Fig. 5, about 73% in the urban area supported such an initiative compared to 55% in the rural area. For the rural area, lower enthusiasm for government intervention were linked to their familiarity with a contributory mechanism and had expressed support for the same over a non-contributory arrangement.

The rural area also showed higher support (about 35%) for a mix of funding involving the government and individuals who can afford to pay compared to about 19% in the urban area who had a similar view. About 9% from both study sites agreed that everyone should pay for health care, which suggests no concern for those who cannot afford to pay. The differences between rural and urban areas in the choices regarding who should pay for health were significant (*P* = 0.001).

The concern for people who cannot afford to pay for health care is clearly demonstrated in Fig. 6 where 38% overall (rural = 31% and urban = 45%) agreed that the very poor should not pay for health care.

The results also indicate that other groups who should possibly be exempted from paying for health care include the disabled, children and the elderly. However, pregnant women received the least support from those to be excluded (rural =0.8% and urban = 7.6%). This was because any pregnant woman who is unable to pay for health care would be poor and the poor are already proposed for exemption. Similar findings were expressed in the qualitative component of the study where there was strong support for full subsidies for those who are too poor to pay. This was expressed in the following statement: “*For me, any Kenyan who cannot pay for health care because they are poor should be supported to get the care that they need…it is that simple…”* (KI3, rural).

For most study participants particularly in the urban area, the government should be the one paying for health care for everyone, arguing that the amount of money inefficiently used every day is enough to provide health care for all. These are illustrated in the following sentiments:

1. *“Every day in the media, you hear a lot of stories about government ministers and senior officials stealing money. If this rampant theft is stopped the government will save enough money to pay for health care for all of us in this country”* (KI11, Urban)
2. *“Everyone is hungry in this country but not people such as the ones employed to manage our funds…. Between a government officer and me, a hungry man, who should steal from whom? It happens the opposite way so we can never pay any money to the government”* (KI7, Urban).

Asked whether they were aware that government funding of health care for all would mean increased taxes, the general feeling was that such taxes had to be quite minimal and increases should be implemented only after the government implements governance measures such as efficient use of resources and eliminating corruption from the public sector.

Study participants were categorical that given the political context, it would be quite difficult to implement a contributory financing system for UHC. This was expressed in three FGDs in the urban site. Participants argued that sharp political divisions current in the country could make a national contributory scheme difficult to implement because of the seemingly low levels of national integration. A participant explained: *“I am a Kenyan; I was born here and know this country very well. There are rich counties and very poor counties and these may not like each other for purely political and ethnic reasons. If people are going to be told that their contributions could be used anywhere in the country, you will see how politicians twist everything into a mess. So to me a predominantly tax-based system would the best way forward”* (Female, FGD1 Urban).

### Design element 3: Benefit package

As shown in Table 1, four scenarios were presented to study participants during the survey in which they were asked to choose one scenario that they felt was most appropriate and affordable for them.

**Table 1:** Broad design of a benefits package

Choice of scenarios	Rural (%)	Urban (%)	Total (%)
Scenario 1: Status quo with current NHIF rates for comprehensive inpatient care at public facilities only	63 (48.8)	75 (23.2)	138 (30.5)
Scenario 2: Comprehensive coverage for outpatient and inpatient services in public sector health facilities but with increased prepayment either at proposed NHIF rates (KSh 500 per month) or increased taxes	45 (34.9)	165 (50.9)	210 (46.4)
Scenario 3: Choice to use private health facilities but pay most of the bill out-of-pocket or from a private insurance arrangement	10 (7.6)	71 (21.9)	85 (17.9)
Scenario 4: None of the above	11(8.5)	13(4.0)	24 (5.3)
Total	129 (100)	324 (100)	453 (100)

Table 1 shows that 46% of informal sector workers would prefer tax increases or the proposed NHIF premium rate of KSh 500 per month if that would accord them access to needed outpatient and inpatient services at public sector health facilities. However, the majority (51%) of those who preferred such an option were in the urban area compared to 35% in the rural area. The likely reason is that urban informal sector workers are economically better off than their rural counterparts as shown in findings elsewhere in the larger study. About 31% in total would stick with an inferior benefit package not because it meets their health needs but most likely they cannot afford other options. The differences in the choices of the benefits package between the two study sites were statistically insignificant (*P* = 0.91).

### Design element 4: Accountability measures in the management of prepaid funds

The possibility of mismanagement of funds for UHC was one of the major concerns among informal sector workers who would want to prepay for health care. From mainly focus group discussions, the areas that needed improvement in terms of managing a prepaid health system included the following: the type of institution to manage the funds and how the head of such an institution is appointed as well as transparency particularly with financial information.

These critical areas for transparency, according to informal sector workers, were followed up in the survey. In terms of preferred institution to manage prepaid funds, there were significant differences between urban and rural study sites (*P* = <0.001). In Table 2, the rural area preferred, by about 56%, that the institution to manage prepaid funds should be semi-autonomous (part government-part private) such as the NHIF. On the other hand, the single largest group in the urban area (about 41%) preferred the government to fully manage such an institution compared to 30% of the rural informal sector that preferred management by the government. The least preferred institutions for each site were a private entity in the rural area (14%) and a semi-autonomous state organ in the urban area (24%). Overall for both study sites, the national government was the most preferred institution (38%) to manage prepaid funds followed by a private entity (29%). Considering the two most preferred institutions in order of priority for each study site, the rural area preferred a semi-autonomous organ and the national government and the urban preferred the national government and a private institution.

**Table 2:** Most preferred institution to manage prepaid funds for universal coverage

Type of institution	Rural (%)	Urban (%)	Total (%)
Government	39 (30.2)	133 (40.8)	172 (37.8)
Private institution	18 (14.0)	115 (35.3)	133 (29.2)
Semi-autonomous state organ	72 (55.8)	78 (24.0)	150 (33.0)
Total	129 (100)	326 (100)	**455 (100)**

Pearson chi^2^ (45.60); *P* = <0.001

Bold data signifies differences between urban and rural sites in their preferences for an institution to manage prepaid funds

## Discussion

At least on average, the majority of informal sector workers prefer a non-contributory financing design as the dominant financing strategy for UHC. However, an overwhelming majority of rural informal sector workers is in support of the contributory system as the dominant approach. The Government of Kenya has stated its desire to expand coverage through social insurance contributions which seems to be the financing strategy of choice for the rural area but not for the urban area. Despite the differences in the choice of the dominant financing strategy for UHC, both rural and urban study participants expressed high levels of mistrust towards the government. Fears of mismanagement of funds as well as political interference and divisions, were highlighted as potential bottlenecks in designing and developing effective prepayment mechanisms for UHC.

Despite the stated bottlenecks, there were strong justifications for a non-contributory financing mechanism to be the main financing approach for UHC. The reasons put forward included the fact that such a system, compared to social insurance contributions, would be more equitable, affordable, easy to implement and sustainable. Such a system was also viewed as inclusive hence elimination of such issues as defaulting payment and dropping out of coverage, meaning that fund flows are likely to be predictable to support UHC. A number of authors [2, 3, 19, 20] have expressed similar views and are supportive of financing UHC through a non-contributory mechanism in contexts with large informal sector populations.

The inclusivity of a non-contributory financing strategy embodies institutional universalism which makes the redistribution process more effective than under a contributory system where population coverage is often gradual beginning with the formal sector. McIntyre [21] and Sachs [22] state that institutional universalism brings about entitlement to services on the basis of citizenship as opposed to targeting. However, this entitlement requires a high level of collective responsibility to facilitate allocation and redistribution of resources to ensure equitable and efficient delivery of services of the same range and quality [23, 24].

An important observation in support for a non-contributory approach to UHC focused on the political economy of expanding coverage in a setting where social cohesion is not strong and therefore the high degree of collective responsibility required for effective redistribution is compromised. In such a setting, the findings indicated, a non-contributory approach to UHC is less likely to accentuate ethnic/regional division current in Kenya where wealthier regions would find it difficult to participate in a redistributive platform. There is evidence that UHC is a less likely scenario in societies with low social solidarity including divisions along ethnic and religious lines, and high income inequalities [25]. Social solidarity is critical in sustaining redistributive policies such as UHC [25, 26]. Without social solidarity, it is difficult to implement policies that require popular support, particularly those that require certain resources from some groups to support others. The political acceptability and sustainability of a financing system for UHC needs to be seriously considered in the design of future prepayment system. However, there are some level of social and political development in Kenya that could help in establishing UHC as a realistic political goal. The implementation of social welfare programmes such as free primary care and free maternity health are important milestones in the country’s quest for UHC. Stuckler et al. [25] explain that expansion of health care coverage normally occurs as part of a broader process of increasing social welfare programmes such as indicated.

The issue of affordability was a key factor in the design preferences of a future prepaid health system. Affordability is not simply having or not having money to purchase health care but also the type of financing mechanism as well as the timing of contributions if in a contributory system. The non-contributory mechanism was viewed as more affordable especially to the poor and other low-income groups because they are not obligated to pay if they do not fall within the taxable income bracket or do not consume ‘vatable’ products.

For the contributory mechanism, there is substantial evidence suggesting that many developing countries with large informal sector populations that have attempted to expand coverage through contributory mechanisms have experienced difficulties. Tangcharoensathien et al. [27] observe that implementing contributory schemes among large informal sector populations is not feasible because of the difficulties in collecting premiums and high administrative costs. Although Rwanda has often been given as a success story pursuing a contributory system, the system is heavily donor dependent and questions have been raised about its sustainability. For example, enrolment to the mutuelles dropped from about 90% in 2012 to 79% in 2017 [28].

In terms of affordability, the contributory mechanism may not be affordable to many low-income earners because contributions are either flat-rated [29] or the structure/frequency of payments are unfavourable to low-income groups. Kenya’s public insurer, the National Hospital Insurance Fund (NHIF) has instituted reforms allowing informal sector workers to pay their monthly premiums in up to three instalments which could resolve some of the unfavourable terms of contributions. The difficulty of prepaying yearly lump-sum premiums has been recorded in Rwanda where timing of contributions coincided with other household obligations such as school fees which together made yearly contribution expensive, leading to defaults in payments [30]. This means that even among those who indicate that they can afford yearly premiums there are those who will default because of other urgent household obligations. To avoid such a scenario, a non-contributory approach to financing UHC would be the most appropriate for a setting such as Kenya.

In terms of management, the proposal for a semi-autonomous entity and the national government by rural and urban informal sector workers respectively, to manage a prepaid system, was somewhat surprising because public sentiments largely perceive both the government and the NHIF as corrupt and untrustworthy with public funds. In the view of the researcher drawn from contextual experience, the choice of government and an entity such as the NHIF were to some extent driven by compromise. For instance, the rural area had already indicated their preference for a contributory system so an institution moulded along the lines of the NHIF would clearly be their choice because a private entity would likely drive up costs. Likewise, the urban area, as demonstrated earlier, strongly preferred a non-contributory system so their choice of the government as the manager of prepaid funds was not unexpected. There were however, strong suggestions for accountability to ensure that the management of funds for UHC remain transparent including public declaration of financial statements, service costs and service entitlement as well as measures taken to prevent fraud.

Finally, drawing from the negative governance culture as perceived by most informal sector workers, it would be difficult to expand coverage whether under contributory or non-contributory approach. A number of reports [31–33] suggesting financial impropriety in the country are likely to make people reluctant to buy into government programmes. In Ghana for example, reports of widespread mismanagement of funds meant for health care eroded trust and social solidarity to make people reluctant to prepay for health care [34, 35]. Therefore, the suggestion by informal sector workers that governance systems need to be more efficient in controlling wastage and fraud need to be seriously considered as an important step toward raising revenues for a non-contributory approach to financing UHC in many LMIC. The European Union and the African Union [36] joint report on social protection suggests that controlling corruption among other measures would improve fiscal space to advance coverage for informal sector populations in Sub-Saharan Africa. The WHO [1] has also urged countries to effectively and efficiently use resources at their disposal to facilitate progress towards universal coverage.

## Conclusion

There was a clear understanding among study participants of the concept of prepaying for health care and why this is important. Why such clarity of information about prepaying for health care had not translated into actual prepayment participation by informal sector populations was linked to several factors including the choice of a prepayment mechanism which emphasises premium payments and is difficult to enforce in law, as well as a number of social, economic and political factors that require interventions sometimes from outside the health system. The need for an acceptable prepayment mechanism for the informal sector including non-contributory financing, a benefit package that is affordable and aligned to the health needs of the population, and accountability measures that punish corruption would go a long way in expanding population coverage and attainment of UHC in Kenya.

## Application of research evidence

The evidence from this study has been presented to the Government of Kenya through the Ministry of Health (MoH) in various fora. The findings are also summarized into policy briefs to be shared with all health sector stakeholders in Kenya.

## Additional files

Additional file 1: Questionnaire. Data here is exclusively quantitative. The statistics involved are descriptive. (DOCX 136 kb)

Additional file 2: FGD. Data here is qualitative and involves discussions around the various ways to prepay for health care. (DOCX 17 kb)

Additional file 3: In-depth interviews. This includes individual key informant views on various ways to prepay for health care. *[These tools were meant for a larger study from which this manuscript has been derived]*. (DOCX 16 kb)

## Abbreviations

CBD: Central business district
CBHI: Community-based health insurance
EA: Enumeration area
ERC: Ethical Review Committee
FGD: Focus group discussion
HFS: Health financing strategy
IDI: In-depth interview
ILO: International Labor Organization
KEMRI: Kenya Medical Research Institute
LMIC: Low- and middle-income countries
MoH: Ministry of Health
NHIF: National Hospital Insurance Fund
PSU: Primary sampling unit
SHI: Social health insurance
UHC: Universal health coverage
WHO: World Health Organization
WTO: World Trade Organization
