# Supplementary material for: Extending coverage to informal sector populations in Kenya: design preferences and implications for financing policy
Source: BMC Health Serv Res. 2018 Jan 9;18:13. doi: 10.1186/s12913-017-2805-z (PMC5761094; doi:10.1186/s12913-017-2805-z)
Supplement: Supplementary file 1 — Questionnaire. Data here is exclusively quantitative. The statistics involved are descriptive. (DOCX 136 kb) [file 12913_2017_2805_MOESM1_ESM.docx]

**Tool 3: QUESTIONNAIRE**

**Financial Protection for the Informal Sector Survey Questionnaire**

**District [___] Site number [___|___|___] Economic Unit number [___|____|____]**

**Informal Sector Survey Questionnaire**

**Interview the owner or a worker who is involved in the day-to-day running of the informal economic unit**

**SECTION 0: INTRODUCTION**

| 0.0 Economic Unit No. |  | | | |
| --- | --- | --- | --- | --- |
| 0.1 District | 1. Mathira East [____\|____] 2. Mombasa [____\|____] | | | |
| 0.2 Village | Name: [____\|____] | | | |
| 0.3 Date of interview | **DD** | **MM** | **YY** | |
|  |  |  |  | |
| 0.4 Name of respondent |  | | | |
| 0.5 Name of interviewer/Fieldworker |  | | | |
| 0.6 Start time | _____________________AM/PM | | | |
| 0.7 End time | _____________________AM/PM | | | |
| 0.8 Results of interview (CODE) | 1. Interview completed | | |  |
|  | 1. Refused to participate | | |  |
|  | 1. Partly completed | | |  |
|  | 1. Respondent not found on third visit | | |  |
|  | 1. Economic unit unattended | | |  |
|  | 1. Not eligible | | |  |
| Supervised/Checked by: |  |  | | |
| **FOR DATA ENTRY ONLY** | | | | |
| Entered by: 1^st^ ENTRY | Name:…………………………………………………………………………..  Date:…………………………………………………………………………… | | | |
| Entered by: 2^nd^ ENTRY | Name:…………………………………………………………………………..  Date:…………………………………………………………………………… | | | |

**SECTION 1: CHARACTERISTICS OF THE INFORMAL SECTOR**

**Financial Protection for the Informal Sector Survey Questionnaire**

**District [___] Site number [___|___|___] Economic Unit number [___|____|____]**

| **1. 0 NAME of respondent:**  ***[****If more than one unit/department, interview all unit/department heads****]*** | | |
| --- | --- | --- |
| **1.11** Gender | M=1 / F=2 | [_____\|____] |
| **1.12** Age | [**Codes**] | [____\|_____] |
| **1.13** Are you the head of your household? | Y=1 / N=2 | [____\|____] |
| **1.14** Marital status | [**Codes**] | [____\|____] |
| **1.15** Your highest level of education | [**Codes**] | [____\|____] |
| **1.16** Is this business your main source of income? | Y=1 / N=2 | [____\|____] |
| **1.17 *[****If NO above****]*** What is the main source of income? | [**Codes**] |  |
| **1.18** Employment status? (*Probe with the* codes) | [**Codes**] | [____\|____] |
| **1.19** How many people including children are in your household? | No.________________ | [____\|____] |
| **1.20** How many are aged 5 years and below? | No. |  |
| **1.21** How many out of this number is aged below 18 years? | No.________________ | [____\|____] |
| **1.22** How many are aged above 75 years? | No.________________ | [____\|____] |
| **1.23** Do you singularly own and operate this business? | Y=1 / N=2 | [____\|____] |
| **1.24** How many are employed in this business? | No.________________ | [____\|____] |
| **1.25** What is the business category (*Interviewer to categorise industry*) | [**Codes**] | [____\|____] |
| **1.26** How many sections does your business have? *(Interview* ***head*** *of each section)* | No.________________ | [____\|____] |
| **1.27** Why did you choose this type of business? | [**Codes**] | [____\|____] |
| **1.28** How long have you operated this particular business? | [**Code**] | [____\|____] |
| **1.29** How many other businesses does the owner of this business have? | No.________________ | [____\|____] |
| **1.30** Has any of your businesses collapsed in the last 5 years? | Y= 1 / N=2 | [____\|____] |
| **1.31** Is this business registered? | Y=1 / N=2 | [____\|____] |
| **1.32** What is the **main** reason for non-registration? | [**Codes**] | [____\|____] |
| **1.33** Location of the business (*Interviewer to record without asking*) | [**Codes**] | [____\|____] |
| **1.34** Type of structure ( *Interviewer to record without asking*) | [**Codes**] | [____\|____] |
| **1.35** Who owns this business premises? (*Probe and select from the* **codes**) | [**Codes**] | [____\|____] |
| **1.36** What do you consider as the biggest threat to your business? | [**Codes**] | [____\|____] |
| **1.37** Do you get paid per: DAY (1); WEEK (2); MONTH (3); OCCASIONALLY (4) [*Don’t ask own account business-person or employer*] | [**Codes**] | [____\|____] |
| **1.38** If paid DAILY, how many days a week do you work? | No.________________ | [____\|____] |
| **1.39** In total how much do you spend in a normal month? | [**Codes**] | [____\|____] |
| **1.40** For your economic status, where would you rank yourself in this picture? | Show Card | [____\|____] |
| **1.41** Does this business have an account where profits are regularly saved? | Y=1 / N=2 | [____\|____] |
| **1.42** Size of land under cultivation (acres) | [**Codes**] |  |
| **1.43** Main crop grown | [**Codes**] |  |

**SECTION 2: EXPERIENCES WITH PUBLIC PROVISION**

*Now I would like to know about your experiences in using public health facilities.*

**Financial Protection for the Informal Sector Survey Questionnaire**

**District [___] Site number [___|___|___] Economic Unit number [___|____|____]**

- 1. **Could you please recall the very last time you used a public health facility?**

| 1. Less than one month ago | 1 |
| --- | --- |
| 1. One to Three months | 2 |
| 1. Four to Six months ago | 3 |
| 1. More than 6 months ago | 4 |

- 1. **Was the last use for:**

| 1. Outpatient care? | **1** |
| --- | --- |
| 1. Inpatient care? | **2** |

- 1. **What type of public health facility did you last use?** *[RECORD name of facility]*

| **Type** | **Facility Name** |  |
| --- | --- | --- |
| 1. Dispensary |  | **1** |
| 1. Health centre |  | **2** |
| 1. District hospital |  | **3** |
| 1. Provincial hospital |  | **4** |
| 1. National hospital |  | **5** |

- 1. **I would like to read to you some of the experiences that people who visit public facilities sometimes talk about. You may answer ‘*True’* if you have faced a similar experience or ‘*False’* if you have not.**

| **Q2.4 In the facility that you used** | **TRUE** | **FALSE** |
| --- | --- | --- |
| 1. Services were quick (I did not have to wait too long on the queue) | 1 | 2 |
| 1. All the drugs I needed were available (I did not have to buy prescriptions) | 1 | 2 |
| 1. Health workers treated me with respect | 1 | 2 |
| 1. The facility was clean including its environs and bed-linen | 1 | 2 |
| 1. I did not have to bribe the health worker to get quick and good treatment | 1 | 2 |
| 1. The health worker explained to me the nature of my illness and gave me appropriate treatment | 1 | 2 |
|  |  |  |
| 1. I had no transport problems because the facility is near my home | 1 | 2 |

**Financial Protection for the Informal Sector Survey Questionnaire**

**District [___] Site number [___|___|___] Economic Unit number [___|____|____]**

- 1. **Based on the experiences that you and other people you know have had at public health facilities, could you choose THREE key areas in order of priority, which you would like to be improved to make services better for everyone? *[****Let respondent list the THREE issues and mark with numbers 1 – 3****].***

| **PROBLEM** | **THREE KEY ISSUES** |
| --- | --- |
| - 1. Waiting times should be reduced |  |
| - 1. Necessary drugs should be available at all times |  |
| - 1. Health workers should handle patients with respect |  |
| - 1. The facility need to be kept clean both outside and inside |  |
| - 1. No health worker should be given any un-receipted payments to offer treatment |  |
| - 1. The health worker should explain to the patient the nature of their illness |  |
| - 1. Facilities should be nearer to the population |  |
| - 1. Other (specify) |  |

- 1. **Have you ever heard of the NHIF?**

| **Yes** | **No** | *[If* ***NO****, move to* ***Q*** ***2****.****9;*** *If* ***YES*** *continue with* ***Q2.7****]* |
| --- | --- | --- |
| **1** | **2** |  |

- 1. **Are you a member of the NHIF?**

| **Yes** | **No** | *[If* ***NO****, move to* ***Q2.9;*** *If* ***YES*** *continue with* ***Q2.8****]* |
| --- | --- | --- |
| **1** | **2** |  |

- 1. **Please tell me TWO MAIN reasons why you are not a member of the NHIF** *[Tick any TWO mentioned by respondent]***:**

| **Q2.8. Reasons for non-membership** | |
| --- | --- |
| - 1. Premium rates are unaffordable | 1 |
| - 1. I fear funds would be mismanaged | 2 |
| - 1. NHIF does not offer outpatient services | 3 |
| - 1. I don’t know where to register and pay premiums | 4 |
| - 1. NHIF offices are too far away | 5 |
| - 1. It is voluntary to join NHIF | 6 |
| - 1. Paying before one is ill is an invitation for illness | 7 |
| - 1. No need to be a member because I’m never ill | 8 |
| - 1. Other (specify) | 9 |

**Financial Protection for the Informal Sector Survey Questionnaire**

**District [___] Site number [___|___|___] Economic Unit number [___|____|____]**

**SECTION 3: CONTRIBUTION MECHANISMS**

*Now I would like to ask you a few questions regarding paying for health care in advance and how, in your opinion, such payments could be done.*

- 1. Who, in your opinion, should pay for health care in this country? *[Let the respondent answer but if there is no response, probe by reading out the options below for the respondent to pick ONE]*

| Q3.1 Who should pay for health care? | CHOICE |  |
| --- | --- | --- |
|  |  |  |
| - 1. The government alone should pay for health care for everyone | 1 |  |
| - 1. The government and those who can afford should pay for health care on behalf of everyone | 2 |  |
| - 1. Everyone should pay for health care | 3 |  |
| - 1. I do not know who should pay for health care | 5 |  |

- 1. *The government does not have enough money to pay for everyone’s health and is encouraging everyone to raise extra money and pay for health care in advance. Payments made in advance for health care are meant to take care of future illness costs so that no one has to pay anything at the hospital when they are ill. Payment in advance can be done in two ways: (1) Pay an additional tax to the government and then the government uses this extra money to pay for health care for everyone; (2) Contribute to a separate organization that collects money from all Kenyans and pays for their treatment costs whenever they fall ill.*

**If everyone were to help in raising extra money to pay for health care, how would you like to raise this money?** *[Read out the two options to the respondent]*

| Q 3.2 Would you prefer: | |  |
| --- | --- | --- |
|  |  |  |
| - 1. To pay additional tax to enable the government to pay for health care for everyone | 1 |  |
| - 1. To contribute directly into a separate organisation that pays for health care for everyone | 2 |  |
| - 1. Both ways are appropriate | 3 |  |
| - 1. Neither of the above is appropriate | 4 |  |

**Financial Protection for the Informal Sector Survey Questionnaire**

**District [___] Site number [___|___|___] Economic Unit number [___|____|____]**

- 1. **Looking at your community, what kinds of people do you feel should NOT pay towards the extra money needed to pay for health care in advance for everyone?** *[Mark ALL the groups that the respondent mentions as people who should not pay for health care and probe for more].*

| Q3.3 Who should NOT pay for health care? | CHOICE |  |
| --- | --- | --- |
|  |  |  |
| - 1. People who are proven to be very poor | 1 |  |
| - 1. Children and the elderly populations | 2 |  |
| - 1. Pregnant women | 3 |  |
| - 1. All disabled people | 4 |  |
| - 1. Other (specify) | 5 |  |

- 1. **In every community, there are usually poor people and others that are better off** *(Show card and point out the different classes of people)*. **With these groups of people in mind, if all were to pay for health care, how much should each group pay?** *[Show respondent Card on contributions levels and read out the corresponding options]*

| **Q3.4 How much *should the different groups of people pay for health care*?** |  |
| --- | --- |
| - 1. Everyone pays the same amount | 1 |
| - 1. Very poor people should not pay at all | 2 |
| - 1. All must pay something but pay according to their levels of income | 3 |

- 1. **By contributing for health care directly into the extra fund, people may prefer to pay their contributions in ways that they feel are easiest for them. Are you aware of any ways that you feel should be used to pay contributions?** *[If no response, probe by reading out the options below for the respondent to choose TWO preferred options].*

| **Q3.5 TWO most convenient ways to pay premiums** | |
| --- | --- |
| - 1. Use mobile phones to pay contributions (M-Pesa) | 1 |
| - 1. Buy scratch cards (similar to those of mobile phones ) and use these to pay contributions | 2 |
| - 1. Use agents at local shops or within the area to pay contributions | 3 |
| - 1. Go to town and pay contributions at the offices in the urban areas | 4 |
| - 1. Use local groups such as SACCOs and agricultural societies | 5 |
| - 1. Other (Specify) | 6 |

**Financial Protection for the Informal Sector Survey Questionnaire**

**District [___] Site number [___|___|___] Economic Unit number [___|____|____]**

**SECTION 4: SOCIAL SOLIDARITY**

*Now I would like to know how you feel about paying for health care in advance.*

- 1. **I would like to read for you a statement and please indicate whether you** *“Strongly agree; Agree; Disagree; or Strongly disagree”* **with this statement:**

|  | **RATING** | | | |
| --- | --- | --- | --- | --- |
|  | **Strongly agree** | **Agree** | **Disagree** | **Strongly disagree** |
| - 1. I would agree to prepay for health care because even though I may not be sick now, my contributions could help someone else or me in future | 1 | 2 | 3 | 4 |

- 1. **Which of the following statements do you agree with most *(****CHOOSE ONE****)***

| **Q4.2 Statement MOST agreed with** | **CHOICE** |
| --- | --- |
| - 1. Everyone should only be expected to pay for their own health care | 1 |
| 1. I would be willing to pay for health care in advance if it benefits my family only | 2 |
| 1. I would be willing to pay for health care in advance if it benefits my family, friends and neighbours | 3 |
| 1. I would be willing to prepay because I understand my prepayment could contribute to bringing health care benefits to all Kenyans | 4 |

**Financial Protection for the Informal Sector Survey Questionnaire**

**District [___] Site number [___|___|___] Economic Unit number [___|____|____]**

**SECTION FIVE: DEFINING BENEFITS PACKAGE**

- 1. **If you were to pay in advance for health care every month, what would you like your payments to cover? I would like to read out for you some options that you could choose from. Please choose one that you feel is best for you and dependants and one that you can afford** *[CHOOSE ONE].*

| **Q5.1 SCENARIO** | **CHOICE** |
| --- | --- |
| - 1. You and all dependants are covered for all medical conditions, both inpatient and outpatient (meaning you don’t have to pay anything whenever you or dependant seeks care) but you are required to visit public and faith-based health facilities only. For this you pay a total of Ksh 500 per month or Ksh 6000 per year | **1** |
| - 1. You and all your dependants are covered for all medical conditions but for inpatient care only (no outpatient care); you can seek care in public and faith-based facilities only. For this you pay Ksh 160 per month or Ksh 1920 per year | **2** |
| 1. You and dependants are covered for all medical conditions, both inpatient and outpatient. You can choose to go to any health facility (private, public or faith-based). If you choose to go to an expensive private facility, only a small part of the bill is paid, the rest you pay by yourself. For this you pay Ksh 500 per month or Ksh 6000 per year | **3** |
| 1. None of these choices suits my needs | **4** |

- 1. *(For choice ‘d’ above)***If none of these choices meets your needs, how much can you afford to pay per month or per year to pay for all health problems for you and dependants, both inpatient and outpatient?______________________________________**
  2. **Based on the choice you have made above (benefits package), you might prefer to pay your contributions- once yearly or in small amounts (instalments.) From your point of view, how would you prefer to pay your contributions? Would it be easy for you to pay** *(Read the options and let the respondent pick one)***:**

| Q5.3 Contribution strategies | CHOICE |
| --- | --- |
| - 1. At once every year | 1 |
| - 1. Once every month | 2 |
| - 1. In instalments every two weeks to make up the required monthly total | 3 |
| - 1. In instalments weekly to make up the stated monthly total | 4 |
| - 1. In instalments daily to make up the total amount required per month | 5 |
| - 1. In instalments any time there is money available to make up the monthly totals | 6 |

**Financial Protection for the Informal Sector Survey Questionnaire**

**District [___] Site number [___|___|___] Economic Unit number [___|____|____]**

**SECTION 6: PREDICTING FINANCIAL POTENTIAL TO SUSTAINABLY PREPAY FOR HEALTH CARE**

*People have different financial abilities and there are groups within the informal sector who have some money and can afford to pay for health care in advance. I would like to seek your opinion regarding what issues to look out for in order to know about people in the informal sector who can afford to raise money to pay for health care in advance. I would like to read to you a list of things that may or not tell whether someone in the informal sector (including farmers) has the ability to prepay for health care. Simply respond by saying whether you* ***‘Strongly Agree; Agree; Disagree, or Strongly Disagree’*** *with what I read out.*

- 1. **Non-agricultural informal sector enterprises** (URBAN area & RURAL market ONLY)**:**

| **Q6.1 [URBAN and non-agricultural enterprise owners/managers]**  **Predictors of financial potential in Urban areas** | **RATING** | | | |
| --- | --- | --- | --- | --- |
|  | Strongly Agree | **Agree** | **Disagree** | **Strongly Disagree** |
| - 1. Type of structure (permanent/temporary/no structure) can tell that an enterprise owner has ability to prepay for health care | 1 | 2 | 3 | 4 |
| - 1. Any enterprise that is licensed is likely to raise enough money to prepay for health care | 1 | 2 | 3 | 4 |
| - 1. An enterprise that employs one or more people generally shows ability to prepay for health care for the owner | 1 | 2 | 3 | 4 |
| - 1. Only business owners in legally allocated areas may have ability to prepay for health care | 1 | 2 | 3 | 4 |
| - 1. A business that occupies more than one room or a large space is able to prepay for health care | 1 | 2 | 3 | 4 |
| - 1. Businesses whose owners are members of a health scheme, a savings scheme (SACCO) or merry-go-round have ability to prepay for health care | 1 | 2 | 3 | 4 |
| - 1. Business owners who spend money on running costs such as employee wages, electricity, telephone and fuel can afford prepaid health care | 1 | 2 | 3 | 4 |
| - 1. Any enterprise owner with other businesses is likely able to prepay for health care | 1 | 2 | 3 | 4 |
| - 1. Gender of the business owner may indicate ability to prepay for health care | 1 | 2 | 3 | 4 |

**Financial Protection for the Informal Sector Survey Questionnaire**

**District [___] Site number [___|___|___] Economic Unit number [___|____|____]**

- 1. Predictors of financial potential in a highly productive agricultural rural setting

| 1. Q6.2 [RURAL AREA ONLY]   Predictors of financial potential in the Rural area (farmers) | RATING | | | |
| --- | --- | --- | --- | --- |
|  | Strongly Agree | Agree | Disagree | Strongly Disagree |
| - 1. Being a casual labourer could mean inability to prepay for health care | 1 | 2 | 3 | 4 |
| - 1. People who own no land are likely to be unable to prepay for health care | 1 | 2 | 3 | 4 |
| - 1. Households with many children and elderly people cannot afford to prepay for health care | 1 | 2 | 3 | 4 |
| - 1. Those living in rented dwellings in the village are too poor to prepay for health care | 1 | 2 | 3 | 4 |
| - 1. Informal sector workers whose children are often out of school for lack of money cannot afford to prepay for health care | 1 | 2 | 3 | 4 |
| - 1. Those who constantly miss some meals each day cannot prepay for health care | 1 | 2 | 3 | 4 |
| - 1. People who do not belong to a prepaid scheme or SACCO or merry-go-round cannot afford to prepay for health care | 1 | 2 | 3 | 4 |
| - 1. The size of land under cultivation is a sign of ability to prepay for health care. | 1 | 2 | 3 | 4 |
| - 1. The number of dairy cows is a sign of ability to prepay for health care. | 1 | 2 | 3 | 4 |

**Financial Protection for the Informal Sector Survey Questionnaire**

**District [___] Site number [___|___|___] Economic Unit number [___|____|____]**

**SECTION 7: MANAGEMENT OF COLLECTED FUNDS**

- 1. **If you were to recommend how to manage all the extra money collected to pay for everyone’s’ health costs, what kind of body would you like to manage this money to use it to buy health services for everyone?** *[If no response, probe by reading out the options and then let the respondent choose ONE].*

| **Q7.1 Type of organization** | **CHOICE** |
| --- | --- |
| - 1. The national government | **1** |
| - 1. A body that has no connection with the government at all | **2** |
| - 1. An organization with some connection to the government but has full control of the funds | **3** |
| - 1. Other (Specify) | **5** |

- 1. **How should the head of this organization holding the money be selected?**

| **Q7.2 Selection strategy** | **CHOICE** |
| --- | --- |
| - 1. Everyone should participate in electing the head of the organization | **1** |
| - 1. Either the president or the minister for health should appoint the person to head the organization | **2** |
| - 1. The head of the organization should be interviewed and selected by an independent panel | **3** |
| - 1. Other (specify) | **4** |

**Financial Protection for the Informal Sector Survey Questionnaire**

**District [___] Site number [___|___|___] Economic Unit number [___|____|____]**

- 1. **By paying for health care in advance, people may need some information concerning the use of their money.** *Please, state whether the kind information that I’m going to read is “****Very*** ***important****;* ***Important****;* ***Somewhat important*** *or* ***Not important****” to you.*

| **Q7.3 Is information on:** | **Very Important** | **Important** | **Somewhat Important** | **Not Important** |
| --- | --- | --- | --- | --- |
| - 1. Amount of money received and used over a period of time | 1 | 2 | 3 | 4 |
| - 1. The range of services on which money was spent | 1 | 2 | 3 | 4 |
| - 1. Measures to avoid fraud and other forms of corruption | 1 | 2 | 3 | 4 |
| - 1. Quality of care at health facilities | 1 | 2 | 3 | 4 |
| - 1. What to expect at health facilities so that patients are empowered to demand better services | 1 | 2 | 3 | 4 |
| - 1. Challenges facing the organization and what is being done to resolve them | 1 | 2 | 3 | 4 |
| - 1. Key appointments made and the procedure for such appointments | 1 | 2 | 3 | 4 |
| - 1. How health facilities are chosen to provide services | 1 | 2 | 3 | 4 |
| - 1. Other *(probe for any other ‘****Important’*** *or* ***‘Very important’*** *information)* | 1 | 2 | 3 | 4 |

- 1. **How would you prefer the organization holding your money to get you the information that you need?** (*Choose* ***TWO*** *most preferred channels*):

| **Q7.4 Preferred information source** | **CHOICE** |
| --- | --- |
| - 1. Radio | **1** |
| - 1. Community leaders and local authorities | **2** |
| - 1. Health workers | **3** |
| - 1. Newspapers | **4** |
| - 1. Community notice-boards | **5** |
| - 1. Other (specify) | **6** |

| **SECTION 8. SOCIO-ECONOMIC STATUS AND ASSET INDEX**  **Financial Protection for the Informal Sector Survey Questionnaire**  **District [___] Site number [___\|___\|___] Economic Unit number [___\|____\|____]** | | | |
| --- | --- | --- | --- |
| **Q8.1** | **SOCIO-ECONOMIC STATUS** | | |
|  | Walls of the main dwelling | 1. Stone 2. Brick/Blocks 3. Mud 4. Wood 5. Cement 6. Iron sheets   7. Other (specify) | [____I____] |
|  | Roof of the main dwelling | 1. Iron sheets 2. Tiles 3. Concrete 4. Makuti 5. Grass 6. Tin 7. Other (specify) | [____I____] |
|  | Floor of the main dwelling | 1. Cement 2. Tiles 3. Earth 4. Other (specify) | [____I____] |
|  | Does your household own the house? | 1. Owns 2. Rented 3. No rent, doesn’t own | [____I____] |
|  | If house is rented, how much is paid on rent? | **Ksh**.________________________ |  |
|  | Main source of drinking water | 1. Piped into the house 2. Piped within the compound 3. Public tap 4. Well/Bore hole 5. River/Stream/Dam 6. Other (specify) | [____I____] |
|  | Toilet facility | 1. Flush toilet 2. Modern pit latrine 3. Traditional pit latrine 4. None | [____I____] |
|  | Main source of cooking fuel | 1. Firewood 2. Kerosene 3. Electricity 4. Gas 5. Charcoal 6. Other (specify) | [____I____] |
|  | Main source of lighting | 1. Kerosene 2. Electricity 3. Solar 4. Other (specify) | [____I____] |
|  | Has your household ever faced food shortages for lack of money? | Yes  No | [____I____] |
|  | | | |

**Financial Protection for the Informal Sector Survey Questionnaire**

**District [___] Site number [___|___|___] Economic Unit number [___|____|____]**

| **Q8.2 ASSET OWNERSHIP** | | | |
| --- | --- | --- | --- |
| **Does your household own:** | | | |
|  | Land | Y =1 N=2 DK=99 | [____I____] |
|  | Television | Y =1 N=2 DK=99 | [____I____] |
|  | Video/DVD | Y =1 N=2 DK=99 | [____I____] |
|  | Fridge/Freezer | Y =1 N=2 DK=99 | [____I____] |
|  | Cookers (Electric/Gas) | Y =1 N=2 DK=99 | [____I____] |
|  | Motorbike | Y =1 N=2 DK=99 | [____I____] |
|  | Vehicle | Y =1 N=2 DK=99 | [____I____] |
|  | Livestock (Cattle, sheep, goats) | Y =1 N=2 DK=99 | [____I____] |

**THANK THE RESPONDENT FOR THEIR TIME AND INQUIRE IF THEY HAVE ANY ISSUES/QUESTIONS TO RAISE.**

**CODES**

**Q1.17 Main source of income**

1. Formal job

2. Other business

3. Spouse

4. Remittance

5. Water vending

6. Farming

7. Other (specify)

**Q1.18**

**Employment status**

1. Employee full time

2. Temporary/Casual

3. Employer

4. Owner

5. Household worker

6. Partnership

7. Unpaid family worker

8. Unpaid apprentice

9. Other (Specify)________

**Q1.15**

**Education level**

1. None

2. Nursery/pre-school

3. Primary

4. Secondary

5. College (mid-level)

6. University

7. Other (specify)

99. Don’t Know

### Q1.14

### Marital status

1: Married

2: Single

3: Divorced /separated

4: Widowed

**Q1.12**

**Age in years**

1. 15<17
2. 18-<24
3. 25-<35
4. 36-50
5. 51+
6. Don’t Know

**Q1.27**

**Why this particular business**

1. Trained in it

2. Family trade

3. Could not find formal employment

4. Market demand

5. Better income

6. Influenced by others

7. Easy to do/start

8. Other (_____________)

**Q1.33**

**Location**

1. Commercial premises

2. Industrial area

3. Open market

4. Jua-kali site

5. Mobile

6. Roadside/Street pavement

7. Residence

8. Farm

9. Other (Specify)__________

99. Don’t know

**Q1.32**

**Reasons for non-registration**

1. Not necessary

2. Process too long

3. Expensive fees

4. Cannot afford bribes

5. Pay council daily

6. Other (specify)

99. Don’t know

**Q1.28**

**Years in Existence**

1. <1
2. 1-<5
3. 5-<10
4. 10-<15
5. 15-<18
6. 18-<35
7. Over 35years
8. Don’t Know

**1.25 Industry category**

1. Food vending
2. Cloth & beauty
3. Shop-keeping
4. Hotel & Food kiosks
5. Manufacturing & Craft
6. Transportation
7. Repair & Maintenance
8. Health & Medical
9. Telecommunication
10. Entertainment
11. Construction
12. Farming & Livestock
13. Energy
14. Stationery
15. Others (specify)

**Q1.35**

**Occupancy of premises**

1. Own

2. Lease

3. Rented

4. Temporary license

5. Free occupation

6. Illegal occupation

7. Other (Specify)__________

99. Don’t know

**Q1.36**

**Main threats to business**

1. Eviction by local or central govt

2. Eviction by owner

3. Natural disasters (fires, rainfall, other accidents

4. Expiry of tenancy

5. Harassment/Bribes from authorities

6. Theft

7. Business competition

8. Low capital

9. Debts

10. Unstable prices of inputs (inflation)

11. Lack of markets

12. Pests

12. Other (Specify)___________

99. DK

**Q1.34**

**Type of structure**

1. Permanent

2. Temporary (fixed)

3. Temporary (movable)

4. No structure

5. Handcart

6. Vehicle

7. Other (Specify)_________

**Q1.39**

**Consumption per month (KSH)**

1. <2500

2. 2500 - 3500

3. 3501 - 4500

4. 4501 - 5500

5. 5501 - 6500

6. 6501 - 7500

7. 7501 - 10000

9. Over 10000

99. DK

**-**

**Q1.24**

**No. employed in business**

1. 1 – 2
2. 3 – 4
3. 5 – 10
4. None (owner only)
5. 11 – 20

**Q1.22**

**No. of people aged >75 years**

1. 1 – 2
2. 3 – 4
3. 5 – 6
4. None

### Q1.21

### No. of children <18 yrs

1. 1 – 3
2. 4 – 6
3. 7 – 10
4. None
5. >10

**Q1.20**

**No. of children <5 yrs**

1. 1 – 3
2. 4 – 6
3. 7 – 10
4. None

**Q1.19**

**Total number of people in household**

1. 1 – 3
2. 4 – 6
3. 7 – 10
4. 11 – 15
5. 16 – 20

**Q3.3**

**Who should not pay extra?**

5. Orphans

6. People with chronic conditions

7. Everyone should pay

**Q7.2**

**How to appoint head of the organization**

4.

**Q7.1**

**Type of Organization**

4.

### Q 5.2

### How much can you afford per year for comprehensive cover

1. 500 – 1000
2. 1001 – 1500
3. 1500 – 2000
4. 2001 – 3000
5. 3001 – 4000
6. 4001 – 5000

**Q3.5**

**Convenient ways to pay**

6. Tax on income

7. Bank deposits

**Q7.3**

**Important information needed by contributors**

**Q1.43**

**Main crop grown**

1. Coffee
2. Tea
3. Garden crops (horticulture)
4. Maize
5. Other (specify)

**Q 1.42**

**Size of farm under cultivation**

1. <1acre

2. 1 – 2 acres

3. 2 – 4 acres

4. 5acres +

### Q 8.1e

### How much paid in rent (KSH)

1. 500 – 1000
2. 1001 – 2500
3. 2501 – 3500
4. 3501 – 4500
5. 4501 – 6000
6. 6001 – 8000
7. 8001 – 10000
8. Over 10000

**Q7.4**

**Ways to disseminate information**

6. Internet

7. Phone sms

**Q1.26**

**No. of Sections in this Business**

1. One
2. Two
3. Three
4. Four
5. Five

**Q2.8**

**Why not NHIF member?**

9. Don’t know the benefits of NHIF

10. It is for formal sector employees only

11. Don’t know cost of premium

12. Long process to join NHIF

13. Never bothered to be a member

### Q 1.38

### No. of days work in a week

1. 1 – 2
2. 3 – 5

6 – 7

**Q1.29**

**How many other businesses owned**

1. One
2. Two
3. Three
4. None
5. Five
6. Six
7. Don’t Know

*\a*

**Tool 4: IN-DEPTH INTERVIEWS: POLICY-MAKERS (NHIF)**

**Health financing situation**

1. What is your opinion about the way health care is currently funded in Kenya?
   - What are the sources of funds?
   - Who decides on the amounts to allocate to the health sector?
   - What services are provided? Are the services provided adequate?
   - Who benefits from the services provided?
2. In your opinion, do you feel that the NHIF has effectively played its part in making health care more accessible to Kenyans?
   - Where has the NHIF succeeded most? Where has it failed most?
   - What needs to be improved?

**Views on universal coverage and priority services**

1. There are some discussions about provision of health care for all citizens (universal coverage). How should this be achieved?
   - In your opinion, what are the possible financing sources for universal coverage?
   - Who should manage funds from each of the sources?
   - In deciding the kinds of services to be provided under a universal system, which services do you regard as most important?

**Prepayment design and financial protection for the informal sector**

1. What is the prospect of a universal system through contributory health insurance schemes such as the NHIF?
   - How can contributory health insurance be made attractive to everyone?
   - If you were to make changes in NHIF, what would you want to change?
   - What should the NHIF do to ensure that the informal sector is more receptive to NHIF membership?
   - Is there a better approach to provide financial protection for the informal sector?
   - What other financing alternatives should be explored to provide coverage to the rest of the population (i.e. those who are not working in the formal or informal sectors)?
   - Could the NHIF learn anything from other health care schemes in the country? Explain
2. What are the current challenges in covering the informal sector?
   - Revenue collection
   - Stability of incomes
   - Trust and solidarity
   - Knowledge and attitudes toward prepaid health care
3. In circumstances where the informal sector is required to pay into a risk pool, how can groups within the sector that are able to prepay for health care be identified so that those least able to pay can be subsidized?
4. What are the possibilities of a tax designed to target the informal sector specifically?

**Purchasing and provision of services**

1. From the mentioned sources of funds, how could the funds be collected and pooled?
   - Should all funds be combined in a single pool or should there be separate pools to cover different groups? Why?
   - Who should purchase health services? Why?
   - Who, in your opinion should provide the services purchased?
   - In your view, how should the providers be paid? Why?

**IN-DEPTH INTERVIEWS: POLICY-MAKERS (MINISTRY OF HEALTH)**

- Tax funding and how to get it to the poor
- Roadmap to UC
- Current funding arrangements
- Funding arrangements to cover the informal sector

**Health financing and policy trajectory**

1. What is your opinion about the way health care is currently funded in Kenya?
   - What are the sources of funds?
   - Who decides on the amounts to allocate to the health sector?
   - What services are provided? Are the services provided adequate?
   - Who benefits from the services?
2. What are the main concerns about health financing in Kenya?
   - What policies are being put in place to address these concerns?
   - What are some of the main highlights of these policies?
   - When do we expect them to be implemented and fully in operation?
   - What are some of the challenges to the implementation of the policies?
   - What kind of support is needed for the policies to become operational?

**Views on universal coverage and priority services**

1. There are some discussions about providing affordable health care for all Kenyans (universal coverage). What is the feasibility of achieving a universal health system?
   - What are the possible financing sources for universal coverage?
   - How should funds from each of the sources be managed?
   - What are the health services that you feel should be covered under a universal system? Why?
   - How should a package of health service benefits be determined?
2. The civil service has its own health insurance coverage, why did the government choose to organize and subsidize coverage for civil servants and not the rest of Kenyans?
   - What would you say about coverage for civil servants? Could it have been done in a different way?
   - Do you foresee all Kenyans getting covered?
3. How is the ministry streamlining health financing in the country to ensure that all Kenyans have access to quality health care in line with the new constitution?

**Prepayment design and financial protection for the informal sector**

1. In your opinion, what financing arrangement can provide effective financial protection to the whole population?
   - Can tax funds be used to provide adequate health care for all?

- What can be done to expand the tax base and the tax revenue collected?
- In your opinion, what should be done to increase amounts allocated to health care from the government budget?
- What are your views about earmarked taxes for health care? Which good/services should be targeted?
- Is meeting the Abuja target (15% of the budget) feasible?
- How can 15% of the budget improve health care in Kenya?
- What do you feel are the challenges to meeting this target?

1. What are the prospects of a universal system through contributory insurance schemes?
   - What kind of scheme is most preferred by the ministry/government? Why?
   - How can the informal sector be covered through such schemes?
   - The current financial protection policies through NHIF do not seem to reach the informal sector, what should be done to improve the situation?
2. Under a universal system, how will the informal sector be covered?
   - In circumstances where the informal sector is required to pay into a risk pool, how can informal sector groups that are able to prepay for health care be identified?
   - What are the possibilities of a tax designed to target the informal sector specifically?
   - What challenges are there in trying to provide financial protection to the informal sector?

**Purchasing and provision of services**

1. From the mentioned sources of funds, how will the funds be collected and pooled?
   - Should all funds be combined in a single pool or should there be separate pools to cover different groups? Why?
   - Who should purchase health services? Why?
   - Who, in your opinion, should provide the services purchased?
   - In your view, how should the providers be paid? Why?
2. What must be done to achieve effective universal coverage in Kenya?
   - How do we ensure that people go to health facilities for treatment instead of relying on poor quality OP clinics, chemists, shopkeepers…?

**IN-DEPTH INTERVIEWS: POLICY-MAKERS (MINISTRY OF FINANCE)**

**Health financing and taxation**

1. What is your opinion about the way health care is currently funded in Kenya?
   - What are the sources of funds?
   - Who decides on the amounts to allocate to the health sector?
   - What services are provided? Are the services provided adequate?
   - Who benefits from the services?
2. In your opinion, can tax funds be used to finance health care for all in Kenya?

- What may be the economic and social consequences of providing health care for all Kenyans through tax funds?
- How does the treasury decide how much should be allocated to health care? Is the health ministry involved in this decision? If so, how?
- What do you think should be done to increase funding for health care?
- What can be done to expand the tax base and the amounts collected? Is there room to improve tax collection methods?
- What is the feasibility of ear-marked taxes for health care? What goods/services can be targeted? Why?
- Are there possibilities of introducing a specific taxation method to target the informal sector?

1. Apart from tax funding, what other funding arrangements should be explored to provide affordable health care for all Kenyans?
   - How can the informal sector be involved in such a system?
   - In circumstances where the informal sector is required to pay into a risk pool, how can informal sector groups that are able to prepay for health care be identified?
   - What challenges are expected in trying to provide financial protection to the informal sector?
2. What challenges exist in efforts to provide adequate and affordable health care for all Kenyans?
   - Political
   - Economic
   - Social
3. How can these challenges be overcome?

**IN-DEPTH INTERVIEWS: POLICY-MAKERS (EXTERNAL AGENCIES)**

**Health financing situation**

1. What is your general opinion about the way health care is currently funded in Kenya?
   - Sources of funds (current funding arrangements)
   - What changes would you like to see?
2. As development partners, where do you see yourself fit in improving the health care financing situation for purposes of universal coverage?
   - What is your opinion regarding health system governance and accountability in Kenya?
   - How can the government improve efficiency in the use of funds and service delivery?
   - Do you foresee an end to external funding for health care?
   - (SWAP)There have been calls to streamline donor funds for health care so that the funds are used as part of government budget for health care rather than for specific health programmes.... How far is Kenya in this regard?

**Views on universal coverage and priority services**

1. With your understanding of the Kenyan context, which is the most feasible policy approach to universal health coverage? Why?
   - Tax funding:
     1. How to expand the tax base
     2. Earmarked taxes for health care
     3. Getting tax funds to the poor
   - Contributory insurance:
     1. Social health insurance
     2. Community-based health insurance
   - Any other arrangement?
2. What do separate health insurance arrangements for civil servants and teachers mean for universal coverage and equity?
   - Is it likely to facilitate universal coverage?
   - Where does such an arrangement leave the rest of Kenyans?
   - Are these private pools within a national insurer?
3. Providing financial protection for the informal sector is a difficult undertaking for many developing countries. How can development partners help Kenya to provide coverage for the informal sector in particular and the rest of the population in general? What policy advice would you give the government for informal sector coverage?
   - Technical capacity
   - Organization
   - Subsidy
4. Not all services can be covered in a universal system, what constitutes the basic benefit package of care from an international perspective?
   - What services do you feel should be prioritized in the Kenyan context?
5. There have been a number of declarations aimed at improving health care, particularly for the poor: Alma Ata; Paris; Abuja, etc.
   - Do we need these milestones?
   - What do you think are the reasons for Kenya’s failure to meet the objectives of these milestones?
6. In your opinion, what lessons can Kenya learn from other countries in terms of health financing and providing coverage for the informal sector?
