# Supplementary material for: Extending coverage to informal sector populations in Kenya: design preferences and implications for financing policy
Source: BMC Health Serv Res. 2018 Jan 9;18:13. doi: 10.1186/s12913-017-2805-z (PMC5761094; doi:10.1186/s12913-017-2805-z)
Supplement: Supplementary file 2 — FGD. Data here is qualitative and involves discussions around the various ways to prepay for health care. (DOCX 17 kb) [file 12913_2017_2805_MOESM2_ESM.docx]

**Tool 1: FOCUS GROUP DISCUSSIONS**

**INFORMAL ECONOMIC ACTIVITIES AND CHALLENGES FACED BY WORKERS**

*For people not employed by the government or private companies, i.e. self-employed people e.g. farmers, small-scale businesses, jua-kali, vegetable vendors and other businesses:*

1. Let us begin by talking about what people in this area who do not have formal jobs do to earn a living:
   - What are the INCOME generating ACTIVITIES in the area?
   - What are their SIZES? (e.g. land, shops, number employed, range of products, etc)
   - Who OWNS the activities (single / group ownership)?
   - What income generating activities have the majority of the area population? WHY?
2. What are some of the major challenges faced by people engaging in the income generating activities that you have mentioned?
   - When are DIFFICULT TIMES to carry out the activities? WHY?
   - Are there months of the year when money is difficult to come by? What happens? What do people do?
   - What PROBLEMS are encountered when carrying out the activities?

**UTILIZATION OF HEALTH SERVICES, PAYMENT FOR CARE AND PRIORITY SERVICES**

1. People have different choices on where to go for treatment or for any other health service. Where do most people in the informal sector around here go for medical services?
   - (What kind of people) Who goes to PUBLIC health facilities; WHY?
   - Who goes to PRIVATE facilities; WHY?
   - Who goes to TRADITIONAL healers; WHY?
   - Of these providers, which one would most people want to go to? WHY?
   - Which are the most commonly used services from these providers?
   - Who in the household decides where to seek treatment?
2. At each of the places where informal sector workers go for health care, what is the MOST COMMON METHOD of paying for the services?
   - What is your opinion about this method of paying for health care? Are there PROBLEMS with this method of payment? Which ones?
   - How else do people pay for health care?
   - How would most people in this area PREFER TO PAY for health care? WHY?
   - Which of these methods of paying for health care would you recommend for people in the informal sector? Why?
3. We discussed the kind of services that informal sector workers receive at the providers that you mentioned:
   - If you were to draw a list of the most important health services for the informal sector, which ones should come first? *Explain*
   - Are these services currently available at each of the providers you mentioned?
   - How do most people in the informal sector pay for these services?
   - How would most people want to pay for these important services?
   - Which health services cost people a lot of money? Why?

**VIEWS HEALTH INSURANCE, WILLINGNESS TO PREPAY FOR HEALTH CARE AND PREFERRED PREPAYMENT DESIGN**

*We have discussed how most people in the informal sector pay for health care. There are different ways of paying for health care including paying out-of-pocket at the point of service and payments made in advance through the government or other organizations. By paying for health care in advance, one receives health services any time one falls ill without having to pay again.*

1. In your opinion, is it a good idea to pay for health care in advance?
   - What sorts of health services do you think the informal sector would like to pay for in advance? Why?
   - In Kenya, there is NHIF that accepts advance payments for health care. Do you know about NHIF? What is good about NHIF? What don’t you like about NHIF?
   - What should be done to get more people from the informal sector to join an organization like the NHIF?
   - What is the maximum that informal sector members would be willing to regularly pay in advance to such an organization?
   - Apart from NHIF, there are small community groups that also pay for health care in advance; do you know of any such groups? What is good about them? What is bad about them?
   - Which do you prefer between an organization such as the NHIF and the small schemes? Why?
   - Would you prefer a large group with many members or a small one? Why?
2. Both NHIF and small community health financing groups (CBHI) are examples of how health care can be paid for in advance. The other method of paying for health care in advance is TAXATION. In taxation, the government collects taxes from people who can pay then uses it to pay for everything including health care, education, military, etc.

- Do you know of any FORMS OF TAXATION that people like you and others pay? How do people pay these taxes?
- Do you think the health sector receives adequate funding from the government? Explain
- What can be done to get more money to the health sector?
- If the government were to increase funding for health care, everyone will need to pay higher taxes. Are you willing to pay more taxes so that more money can be raised for health care for all?
- In what ways would people around here prefer to pay tax?
- Which of these methods of paying for health care in advance (Taxation; NHIF; CBHI) is best for the informal sector? WHY?
- The law may require that everyone pays for health care in advance by making regular payments for future illness costs. What is your opinion about compulsory payments for health care in advance?

*[For payments made in advance for health care, there are two main ways how this can be done:* ***Tax*** *(where a small part of one’s income is paid to the government, and which is then used to pay for health services and other social services like education); and* ***Insurance schemes*** *(where people contribute regular amounts to a group/organization, then the collections are used to pay for health care costs for those who contribute. In health insurance schemes, people may organize themselves at community level; for example, some people in this area may decide to form their own group where they make regular contributions for future health care costs. Alternatively, everyone in this country can get involved in one large health group into which everyone contributes].*

**VIEWS ON UNIVERSAL COVERAGE AND ABILITY TO PREPAY (ATPP) FOR HEALTH CARE**

*If the government together with some donors were to ensure that everyone has access to quality and timely health care, it would require that all of us who have some money make financial contributions to pay for health care in advance. This means that no one will pay for health care from their pockets at the facility. The essence of such a plan is that some would pay but may take a long time before getting ill meaning that people who need more health care but have no money would use other people’s payments.*

1. As members of the informal sector, what do you think of such a plan? How would you like to be involved?
   - Some informal sector workers can support such a plan by making regular contributions to pay for health care in advance. What would you look for so that you can tell that someone self-employed in a business or farming can afford to pay for health care in advance?
   - Among the main informal economic activities that you mentioned, which of them are more likely to make regular payments for health care, i.e. monthly, bi-monthly, etc? WHY?
   - What can make it difficult for self-employed people to pay for health care in advance?
   - Do the informal sector activities bring regular income however small? Which ones have reliable income? Which don’t? Why?

- What do we do with people who are too poor to pay for health care in advance?
- How do we know that they cannot afford to pay for health care?

**RECOMMENDATIONS FOR FUTURE FINANCING**

1. What changes would most people like to have to make paying for health care in advance attractive to the informal sector?
   - What challenges are expected in trying to persuade the informal sector to pay for health care in advance?
