# Supplementary material for: Extending coverage to informal sector populations in Kenya: design preferences and implications for financing policy
Source: BMC Health Serv Res. 2018 Jan 9;18:13. doi: 10.1186/s12913-017-2805-z (PMC5761094; doi:10.1186/s12913-017-2805-z)
Supplement: Supplementary file 3 — In-depth interviews. This includes individual key informant views on various ways to prepay for health care. [These tools were meant for a larger study from which this manuscript has been derived]. (DOCX 16 kb) [file 12913_2017_2805_MOESM3_ESM.docx]

**Tool 2: IN-DEPTH INTERVIEWS AT COMMUNITY LEVEL**

**NATURE OF INFORMAL ECONOMIC ACTIVITIES**

1. There are many income generating activities that people engage in as self-employed or employees if they are not employed by the government, companies or any other formal jobs. Which are the most common income generating activities for people with no formal employment in this area?
   - Which income generating activities do most people engage in?
   - SIZES of the various activities (e.g. land, shops, number employed, range of products, etc)
   - Who OWNS these activities (single / group ownership)
   - What major problems are faced by people engaging in these activities?

**UTILIZATION OF SERVICES, PAYMENT FOR HEALTH CARE AND PRIORITY HEALTH SERVICES**

1. Health care can be sought in different places. Which are the main service providers in this area?
   - What kinds of PUBLIC service providers are available? Who are the main users of these providers? Why?
   - Are there PRIVATE providers? How much do they cost? Who uses them most? Why?
   - What other service providers are in the area? Who uses them most? Why?
   - Among these providers, which one has the majority of people seeking care? WHY?
   - Which providers are MOST PREFERRED by most people here? WHY?
   - How do MOST PEOPLE PAY for health care when they visit these providers?
   - What do you regard as the MAIN PROBLEMS with this method of payment for most people?
   - What OTHER METHODS of payment exist for the rest of the people?
   - Which of these payment methods would you recommend for informal sector workers? WHY?
2. What are the MAIN HEALTH PROBLEMS presented by both children and adults visiting these providers?
   - Are services for these health problems available? Are services SATISFACTORY?
   - Which health services would most people like to have at the health facilities in this area? WHY?
   - What health services are MOST IMPORTANT to women? Children? Men?
   - How do most people pay for these services?
   - Do you think there are problems with this method of payment? Which ones?
   - How would you like to pay for the most important health services? Would you recommend all people to pay for such services the same way?

*[If not clear, explain that health care can be paid for from the pocket at the point of service or prepaid i.e. paid for in advance].*

**VIEWS ON HEALTH INSURANCE, WILLINGNESS TO PREPAY FOR HEALTH CARE AND PREFERRED PREPAYMENT DESIGN**

1. In your opinion, which, between direct (out-of-pocket) payment and payment in advance for health care, is better? WHY?
   - Which organizations accept payments for health care in advance in this area?
   - What kind of people are members of each of these organizations?
   - What is good about an organization such as the NHIF? What don’t most people like about NHIF? What about CBHI?
   - How can we make each of these organizations better for all people to pay for health care in advance?

*[Explain that NHIF is a country-wide organization with many members while CBHI is a small one mainly involving people in a small area]*

- - Which do you think most people in this area prefer between NHIF and the small schemes? Why?
  - What do you think most people here would prefer: a small scheme or a large one? Why?

1. The NHIF would like its members in the informal sector to pay between Ksh300 and Ksh500 per month. Are these rates affordable in this area?
2. You may already know that we can pay for health care in advance through organizations such as the NHIF and through small community health financing groups (CBHI). **Taxation** is a third way of paying for health care in advance, wherein the government collects taxes from people and companies then uses it to pay for everything including health care, education, military, etc.

- Do you know of any FORMS OF TAXATION that people usually pay?

*[Explain various forms of taxes]*

- Do you think the health sector receives adequate funding from the government? Explain
- What can be done to get more money to the health sector?
- If the government were to increase funding for health care, we will need to pay higher taxes. Do you think people in this area would be willing to pay more taxes so that more money can be raised for health care for all?
- What kinds of taxes would be more appropriate for people without formal employment?
- Of the various methods of paying for health care in advance: Taxation, NHIF and CBHI, which do you think can work best for the informal sector? WHY?
- The law may require that everyone pays for health care in advance by making regular payments for future illness costs. Do you believe it is advisable to make it mandatory for everyone to have health care paid for in advance?
- What should be done to encourage people in the informal sector to pay for health care in advance?

*[For payments made in advance for health care, there are two main ways how this can be done:* ***Tax*** *(where a small part of one’s income is paid to the government, and which is then used to pay for health services and other social services like education); and* ***Insurance schemes*** *(where people contribute regular amounts to a group/organization, then the collections are used to pay for health care costs for those who contribute. In health insurance schemes, people may organize themselves at community level; for example, some people in this area may decide to form their own group where they make regular contributions for future health care costs. Alternatively, everyone in this country can get involved in one large health group into which everyone contributes].*

**VIEWS ON UNIVERSAL COVERAGE AND ABILITY TO PREPAY (ATPP) FOR HEALTH CARE**

*By encouraging people to pay for health care in advance, the government would like to make sure that everyone, including the very poor who cannot pay, gets quality and timely health care. This means that people, who can pay some money however small, may help in paying for those who are less able. With this kind of payment, no one will pay for health care from their pockets at the facility.*

1. In your opinion, is it acceptable for most people who contribute to also cater for the health of those who cannot contribute?
   - Who should pay for poor people who cannot afford to pay on a regular basis?
   - How does the community identify these very poor people? What do they have/not have that we consider them as too poor to pay anything?
   - There are people in the informal sector who are able to pay for health care in advance. What can tell that someone self-employed in a business or farming can afford to pay for health care in advance?
   - Among the main informal economic activities that you mentioned, which of them are more likely to make regular payments for health care, i.e. monthly, bi-monthly, etc? WHY?
   - What is likely to make it difficult for self-employed people to pay for health care in advance?
   - Do most self-employed people have regular income, even if low? Which informal economic activities have regular income? Which don’t? Why?
   - How much can most people afford to pay on a monthly basis?
2. What OTHER INFORMATION would you like to share?
